# Attentional bias in individuals with depression and adverse childhood experiences: influence of the noradrenergic system?

**DOI:** 10.1007/s00213-021-05969-7

**Published:** 2021-10-04

**Authors:** Linn K. Kuehl, Christian E. Deuter, Jan Nowacki, Lisa Ueberrueck, Katja Wingenfeld, Christian Otte

**Affiliations:** 1 Charité Universitätsmedizin Berlin, corporate member of Freie Universität Berlin, Humboldt-Universität zu Berlin, and Berlin Institute of Health, Campus Benjamin Franklin, Department of Psychiatry and Psychotherapy, Berlin, Germany; 2 Department of Psychology, Clinical Psychology and Psychotherapy, MSB Medical School Berlin, Berlin, Germany

**Keywords:** Alpha2-adrenergic receptor, Childhood trauma, Dot probe, Major depressive disorder, Norepinephrine, Yohimbine

## Abstract

**Rationale:**

Major depressive disorder (MDD) is a severe mental disorder with affective, cognitive, and somatic symptoms. Mood congruent cognitive biases, including a negative attentional bias, are important for development, maintenance, and recurrence of depressive symptoms. MDD is associated with maladaptive changes in the biological stress systems such as dysregulations of central noradrenergic alpha2-receptors in the locus coeruleus-noradrenergic system, which can affect cognitive processes including attention. Patients with adverse childhood experiences (ACE), representing severe stress experiences in early life, might be particularly affected.

**Objectives:**

With an experimental design, we aimed to gain further knowledge about the role of noradrenergic activity for attentional bias in MDD patients with and without ACE.

**Methods:**

We tested the effect of increased noradrenergic activity induced by the alpha2-receptor blocker yohimbine on attentional bias in a placebo-controlled repeated measures design. Four groups were included as follows: MDD patients with and without ACE, and healthy participants with and without ACE (total *N* = 128, all without antidepressant medication).

**Results:**

A significant effect of MDD on attentional bias scores of sad face pictures (*p* = .037) indicated a facilitated attentional processing of sad face pictures in MDD patients (compared to non-MDD individuals). However, we found no such effect of ACE. For attentional bias of happy face pictures, we found no significant effects of MDD and ACE. Even though a higher increase of blood pressure and salivary alpha-amylase following yohimbine compared to placebo indicated successful noradrenergic stimulation, we found no significant effects of yohimbine on attentional bias of happy or sad face pictures.

**Conclusions:**

Our results are consistent with the hypothesis of a negative attentional bias in MDD patients. However, as we found no effect of ACE or yohimbine, further research is needed to understand the mechanisms by which ACE increases the risk of MDD and to understand the biological basis of the MDD-related negative attentional bias.

## Introduction

Major depressive disorder (MDD) is a serious mental disorder associated with high subjective suffering and pronounced functional disruptions (Ferrari et al. 2013; Otte et al. 2016; Saarni et al. 2007). Among other symptoms, MDD patients suffer from low mood, lack of motivation and cognitive dysfunction. Mood congruent cognitive biases in the processing of emotional information, such as an attentional bias for negative stimuli (Armstrong and Olatunji 2012; Caseras et al. 2007; Mathews and MacLeod 2005; Peckham et al. 2010), are a prominent feature of MDD and have been implicated in the development, maintenance, and treatment of MDD (Beck & Bredemeier, 2016; Joormann and Quinn 2014; LeMoult and Gotlib 2019). Furthermore, MDD patients tend to interpret ambiguous information more negatively compared to healthy individuals (Lawson et al. 2002; Lee et al. 2016; Orchard et al. 2016; Voncken et al. 2007) and to a memory bias for negative information (Bradley et al. 1996; Gaddy and Ingram 2014; Mathews and MacLeod 2005; Power et al. 2000; Taylor and John 2004; Watkins et al. 2000; Watkins et al. 1996; Yang et al. 2016).

Several studies indicate an unfavorable relation of dysregulations in the biological stress systems in MDD and cognitive performance (Behnken et al. 2013; Gomez et al. 2006; Harmer et al. 2009; Hinkelmann et al. 2009, 2013; O'Hara et al. 2007; Schlosser et al. 2011; Wingenfeld et al. 2013), mainly with a focus on cortisol and the hypothalamic–pituitary–adrenal axis and to a lower extend on the locus coeruleus-noradrenaline (LC-NA) system. Since the noradrenergic system has an impact on cognitive processes (Chamberlain and Robbins 2013; Mather et al. 2016; Sara 2016) and attentional biases (Ehlers and Todd 2017), dysregulations of the LC-NA system could play an important role in cognitive dysfunctions associated with MDD. Enhanced binding capacity of central noradrenergic alpha2-receptors, especially in the LC and prefrontal cortex areas, were found in MDD patients (Callado et al. 1998; Cottingham and Wang 2012; Garcia-Sevilla et al. 1999; Goddard et al. 2010; Meana et al. 1992; Ordway et al. 2003; Rivero et al. 2014). Because alpha2-receptors are involved in the autoregulation of NA release, this might lead to lower central NA levels and in turn affect cognitive processes. Described effects of NA activity on cognitive processes include allocation of attentional resources and processing of emotional material, e.g., emotional learning and memory (Giustino and Maren 2018) as well as emotion recognition (Oliva and Anikin 2018) and biased attention based on associative learning processes (Ehlers and Todd 2017). Emotional information is often processed differently from neutral information and depends on the valence of the information and its meaning for the individual. This leads to biased processing of emotional information. This bias also seems to be affected by NA activity. For example, Vasa et al.’s study (Vasa et al. 2009) demonstrated an attentional bias for angry faces following placebo that was vanished following single administration of the NA alpha-2 receptor blocker yohimbine. MDD associated alterations in the LC-NA system might contribute to negative biases in processing of emotional information in MDD patients. First experimental investigations suggest increased sensitivity for noradrenergic stimulation regarding cognitive alterations in MDD. A single administration of the NA reuptake inhibitor reboxetine could reverse negative bias in emotional processing such as emotion recognition and memory of self-relevant information in MDD patients (Harmer et al. 2009). A study from our group (Wingenfeld et al. 2013) demonstrated a positive effect of a single administration of the alpha2-receptor blocker yohimbine on memory consolidation, which was more prominent in MDD patients compared to healthy controls. Again, NA stimulation seemed to compensate for the adverse effects of MDD on cognition. As the effect of NA stimulation on memory was significantly affected by self-reported childhood adversity in the group of MDD patients, alterations in the LC-NA system might be especially pronounced in a subgroup of MDD patients with a history of childhood adversity.

Adverse childhood experiences (ACE) such as emotional neglect, sexual or physical abuse seem to play an important role in the development of vulnerability to depression and about one half of MDD patients have ACE in their biography (Nelson et al. 2017; Nemeroff 2016). On the one hand, ACE represent severe stressors and can contribute to alterations in the biological stress systems (Flugge et al. 2003; Heim and Nemeroff 2002; Ladd et al. 1996; Meaney et al. 1995; Otte et al. 2016; Plotsky and Meaney 1993). Accordingly, some experimental studies have demonstrated a higher NA reactivity to stress in individuals with ACE compared to those without ACE (Heim et al. 2000; Kuras et al. 2017; Otte et al. 2005). On the other hand, ACE have been associated with alterations in cognitive performance later in life (Hedges and Woon 2011; Lovallo et al. 2013; Pechtel and Pizzagalli 2011) including modification of cognitive biases for emotional information (Herzog et al. 2018; Pfaltz et al. 2019; Vrijsen et al. 2015). These ACE-related alterations may contribute to the increased vulnerability to MDD in individuals with childhood adversity. Indeed, a few studies on attentional bias in MDD included ACE in their analysis and found associations between attentional biases for emotional faces and ACE in MDD patients (Bodenschatz et al. 2019; England-Mason et al. 2018; Günther et al. 2015). However, this means, if ACE is not systematically included on studies on attentional bias in MDD, effects of MDD and ACE could be easily confounded. It therefore seems important to include ACE as a factor in the design of such studies.

As mentioned above, a negative attentional bias may contribute to the development, maintenance, and treatment of MDD. It is therefore important to better understand the underlying biological mechanisms of these relationships. In this study, we aimed to investigate whether alterations in the LC-NA system of MDD patients, e.g., in alpha-2 receptor functioning, may contribute to biased attention for emotional information. Our study used an emotional dot probe task as a measure of attentional bias, conducted after administration of the alpha2-receptor blocker yohimbine, which stimulates NA activity, and after placebo in a repeated measures design. We included four groups to implement a study design in which the factors MDD and ACE were fully crossed to disentangle the potential role of ACE: MDD patients with and without ACE, and healthy participants with and without ACE (all without antidepressant medication).

Importantly, MDD often co-occurs with anxiety, and measures of depression and anxiety correlate highly. Although both conditions are characterized by negative affectivity and share overlapping features, both are separate functional entities that can be conceptually differentiated. Depression is generally associated with goal loss and lack of positive affect (anhedonia); depressive processing and cognitive style is biased toward past mistakes and failures and an enhanced focus on the individual’s personal and emotional world. In contrast to this past-oriented, ruminative thinking, anxiety is future-oriented and focused on the prevention of harm and loss (Eysenck and Fajkowska 2018). Previous studies have been able to show an influence on attentional biases for both conditions (Bar-Haim et al. 2007; Kircanski and Gotlib 2015; Klein et al. 2018). However, while anxiety is associated with faster orienting to threatening stimuli such as angry facial expression, depression is characterized by a difficulty in disengaging from dysphoric content (Armstrong and Olatunji 2012). Since we expected higher measures of anxiety for the MDD groups, yet wanted to investigate attentional biases genuinely for MDD (Peckham et al. 2010), we deliberately chose depression-relevant stimuli with happy and sad facial expressions.

We hypothesized (1) an attentional bias toward sad face stimuli and away from positive face stimuli (as measured by the dot probe paradigm) in MDD compared to non-MDD. (2) The effect of MDD on attentional bias should be reduced following intake of yohimbine, due to increasing NA activity, and (3) this reduction should be especially pronounced in interaction with ACE.

## Methods

### Participants

This study was part of a larger research project, which will be and has been reported elsewhere (de Punder et al. 2018; Deuter et al. 2020; Kuehl et al. 2019; Schulz et al. 2021).The study design was approved by the ethical committee of the German Psychological Society to adhere to the Declaration of Helsinki. All participants provided written informed consent. Healthy participants and outpatients received monetary compensation (100 €) for their participation. Patients with MDD and healthy participants were recruited by public postings and from our specialized affective disorder unit at the Department of Psychiatry and Psychotherapy, Campus Benjamin Franklin, Charité—Universitätsmedizin Berlin.

Depressed patients were included if they fulfilled criteria for a current episode of MDD as assessed by a trained psychologist, using a German version of the Structured Clinical Interview for DSM-IV axis I (SCID-I) to validate psychiatric diagnoses (Wittchen et al. 1997). In addition to the SCID-I interview, current depressive symptoms were captured by a clinical rating scale and a questionnaire, the Montgomery Asberg Depression Rating Scale (MADRS) (Montgomery and Asberg 1979; Williams and Kobak 2008) and the Beck Depression Inventory (BDI) (Beck, Steer, & Hautzinger, 1994). ACE was defined as repeated physical or sexual abuse at least once a month over 1 year or more (adapted from Heim et al. 2000) before the age of 18, assessed by a screening interview, and validated by the German version of the semi-structured interview, the Early Trauma Inventory (Bremner et al. 2000; Wingenfeld et al. 2011). Additionally, adverse childhood experiences were measured using a German version of the Childhood Trauma Questionnaire (CTQ) (Bernstein et al. 2003; Wingenfeld et al. 2010). In the MDD groups, schizophrenia, schizoaffective disorder, bipolar disorder, depressive disorder with psychotic features, dementia, abuse of alcohol or drugs, and panic disorder led to exclusion. Healthy participants with and without ACE were free of any current mental disorder. Further exclusion criteria for all participants were CNS relevant diseases, neurological diseases, severe somatic diseases, diabetes type 1 and 2, steroid diseases, hypertonia, current infections, pregnancy, and the intake of psychotropic medication. Physical health criteria were checked by physical examination, clinical interview, and blood test.

The final sample consisted of 128 participants with completed measurements on both days: 27 MDD patients with ACE (MDD + /ACE +), 26 MDD patients without ACE (MDD + /ACE -), 29 participants with ACE but no current or lifetime MDD (MDD - /ACE +), and 46 participants with no current or lifetime MDD and no childhood adversity (MDD -/ACE -).

### Procedure

Diagnostic assessment took place on a separate day prior to the laboratory testing and included psychiatric and medical assessment (for more details, see Kuehl et al. 2020). Participants were tested on two separate laboratory sessions. Experimental setup was identical on both days, except for the oral administration of either yohimbine (10 mg) or placebo in a double-blind, quasi-randomized design. All participants had to refrain from strenuous physical activity and caffeine consumption on the testing days. When they arrived at the laboratory at 09:30 h, participants were seated in a comfortable chair, provided a first saliva sample and blood pressure was measured after a 5 min resting period. Thereafter, participants were orally administered either yohimbine or placebo (09:45 h). Participants then waited 60 min (until 10:45) to allow digestive absorption of the drug.

The dot probe test started at 11:15 h, had a duration of approximately 15 min and was part of an extended study setup, which will be and has been reported elsewhere (Deuter et al. 2020; Kuehl et al. 2020; Schulz et al. 2021). As previous studies demonstrated a plasma peak level of yohimbine at 90 min after oral intake (O'Carroll et al. 1999; Peskind et al. 1995), drug effects were assumed to be present at testing time. The experimental tests had a total duration of approximately 70 min. The total duration of the experimental setup was approx. 2.5 h. The time between the two test sessions was at least 1 day with an average interval of 5.7 days (*SD*: 5.8). The order of interventions according to drug order (yohimbine vs. placebo) and test version (version A vs. version B) was counterbalanced between participants according to group assignment in a quasi-randomized way to minimize order effects.

### Emotional dot probe paradigm

**Fig. 1:**
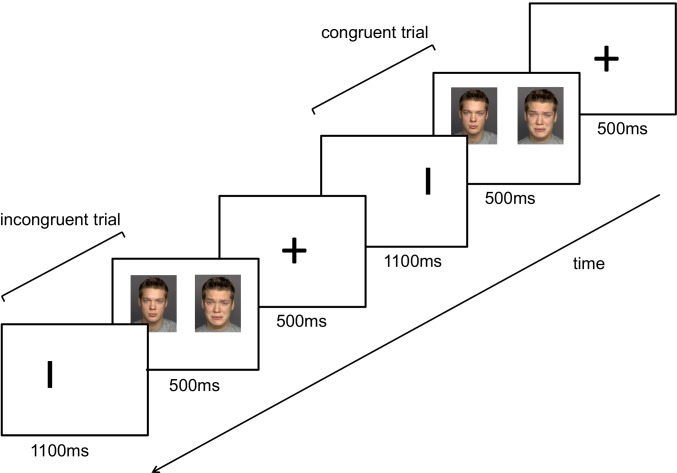
Procedure of the emotional dot probe task: after presentation of a fixation cross (500 ms), a pair of face pictures of the same person occurred (500 ms) followed by a bar (1100 ms). Subjects were asked to respond to the bar with either left or right keystroke according to the position of the bar. In congruent trials, the bar replaced the position of a face picture with an emotional expression (happy or sad), in incongruent trials, the bar replaced the position of a face picture with neutral expression.

For the emotional dot-probe paradigm, we used a computer-based, emotional version of the visual dot-probe paradigm, which is a measure of selective attention toward emotional cues (MacLeod et al. 1986). A set of human faces from the FACES database (Ebner et al. 2010) was used, comprising 20 different persons (10 female, 10 male; different from persons whose pictures served as face stimuli in the approach avoidance paradigm) with happy, sad, and neutral facial expressions. A set of two pictures with human faces was shortly presented on a computer screen (for 500 ms), serving as stimuli. A picture set always consisted of two facial expressions of the same person, either paired as neutral-sad, neutral-happy, or neutral–neutral. One of these pictures was presented on the left side of the computer screen, the other picture on the right side. Afterwards, either the left or the right picture was replaced by a vertical bar which served as the cue (1100 ms). The participants were instructed to pay attention only to the cues and press one of two keys (left vs. right) as fast as possible in reaction to the cue´s position. The attentional capture was reflected in the response latency. It is assumed that participants react faster when the cue replaces the picture at which they antecedently concentrated their attention on. Emotional stimuli are assumed to bind more attention compared to neutral stimuli. In a congruent trial, the cue replaced the location of an emotional stimulus (positive or negative facial expression). In an incongruent trial, the cue replaced a neutral stimulus (neutral facial expression) (please see Fig. 1). In total, 200 trials were presented: 40 trials in the neutral condition, 40 trials in the positive congruent condition, 40 trials in the negative congruent condition, 40 trials in the positive incongruent condition, and 40 trials in the negative incongruent condition. The position of the facial pictures was counterbalanced between left and right, and the order between all trials was quasi-randomized. The total duration of the test was about 15 min. The “attentional bias index” can be determined by calculating the average reaction time for incongruence minus congruence (MacLeod and Mathews 1988; Tsumura and Shimada 2012):

Attentional bias index = ½ [(incogruence right − congruence right) + (incongruence left − congruence left)].

A positive attentional bias index score can be interpreted as an attentional bias toward the emotional stimulus, i.e., attention toward the happy or sad faces and a negative score as an attentional bias toward the neutral stimulus, i.e., avoiding the sad or happy faces.

We used the E-Prime 2.0 presentation software for stimulus presentation and data acquisition. Randomization of task lists was performed using RQube (Seifert and Britz 2007). 

### Physiological measurements

#### Blood pressure measurement

Discrete blood pressure measurements were taken using a standard, automated pressure cuff, which was fixed around the right upper arm (Dinamap 1846 SX). Blood pressure was measured 10 min before intake of yohimbine (pre) and 60, 90, 120, and 150 min after intake of yohimbine (post).

#### Assessment and analysis of salivary alpha-amylase

Six saliva samples were collected on each laboratory session in order to determine salivary-free alpha-amylase (sAA). All patients and participants had to refrain from eating for 2 h before testing and from drinking for 30 min before each saliva sample collection. All participants received instructions on the correct use of the Salivette salivary collection device (Salivettes®, blue cap; Sarstedt AG, Nümbrecht, Germany). Saliva samples were taken 15 and 5 min before intake of yohimbine (pre) and 60, 90, 120, and 150 min after intake of yohimbine (post). All samples were immediately stored on ice and subsequently stored at − 80 °C before biochemical analyses were performed in the Neurobiology Laboratory of the Department of Psychiatry, Charité Universitätsmedizin Berlin. The salivary alpha-amylase activity was determined using a modified protocol of a previously published direct alpha-amylase assay (Lorentz et al. 1999). Inter- and intra-assay coefficients of variation were both lower than 10% for sAA activity.

### Statistical analysis

SPSS version 27.0 was used for all statistical analyses.

Demographic and clinical characteristics, including depression and childhood trauma scores in all subscales of the BDI-II, the MADRS, the CTQ, and the ETI, were analyzed with one-way ANOVA for continuous variables or chi^2^ test for dichotomous variables across groups. Blood pressure and sAA were evaluated using an analysis of variance (ANOVA), with the between-subjects factor “group” (MDD + /ACE + , MDD + /ACE - , MDD - /ACE + , MDD - /ACE -) and the within-subjects factors “treatment” (yohimbine vs. placebo) and “time.” Before analysis, data of salivary alpha-amylase were log-transformed because values were not normally distributed. To analyze differences between groups, Bonferroni corrected post-hoc tests were used.

To analyze the influence of yohimbine administration on attentional bias for happy and sad face expressions, we used 2 × 2 × 2 repeated measures ANOVAs with “treatment” (yohimbine vs. placebo) as within-subject factor and “MDD” (MDD vs. non MDD) and “ACE” (ACE vs. non ACE) as between-subject factor to analyze attentional biases for happy and sad face stimuli separately (Joormann and Gotlib 2007). Attentional bias indices were first calculated from reaction times as described above (see the “Emotional dot probe paradigm” section). Reaction times that were less than 100 ms (anticipation error) or greater than 1500 ms (lack of concentration) were excluded from statistical analyses to reduce the influence of ouliers.

A *p*-value smaller than 0.05 was considered to indicate statistical significance. In the case of violations of sphericity, reported *p* values were Greenhouse–Geisser corrected.

## Results

### Sample characteristics

For demographic and clinical data of the final sample, please see also Table 1.

**Table 1:** Sample characteristics of MDD patients with and without ACE and healthy participants with and without ACE

	MDD + /ACE + *N* = 27	MDD + /ACE - *N* = 26	MDD - /ACE + *N* = 29	MDD - /ACE - *N* = 46	Statistics
Sex (f/m)	14/13	14/12	17/12	24/22	*p* = .95
Age (years; *SD*)	40.0 (11.1)	34.4 (11.0)	33.1 (10.0)	34.9 (10.5)	*p* = .08
Education (years; *SD*)	11.1 (1.6)	11.9 (1.4)	11.7 (1.5)	11.8 (1.5)	*p* = .25
Body mass index (kg/m^2^)	24.9 (2.9)	22.6 (3.3)	23.7 (3.6)	23.4 (3.3)	*p* = .09
Use of hormonal contraceptives (women; y/n)	4/10	4/10	6/11	5/19	*p* = .79
Time since MDD onset (years; *SD*)	17.8 (10.4)	10.7 (8.6)	n.a	n.a	*p* = .01
Age at MDD onset (years; *SD*)	22.3 (11.6)	22.8 (10.2)	n.a	n.a	*p* = .88
Length of current MD episode (months; *SD*)	10.7 (12.5)	11.4 (14.5)	n.a	n.a	*p* = .86
*Depressive symptoms*					
MADRS (*SD*)	27.5 (7.8)	27.8 (6.0)	1.9 (1.9)	0.7 (1.2)	*p* ˂ .001 MDD + /ACE + = MDD + /ACE** - ** > MDD** - **/ACE + = MDD** - **/ACE** - **
BDI (*SD*)	25.9 (8.6)	24.5 (7.2)	4.6 (4.5)	1.6 (1.8)	*p* ˂ .001 MDD + /ACE + = MDD + /ACE** - ** > MDD** - **/ACE + = MDD** - **/ACE** - **
*Adverse childhood experiences*					
ETI (*SD*)	704 (452)	168 (169)	581 (338)	16 (22)	*p* ˂ .001 MDD + /ACE + = MDD** - **/ACE + > MDD + /ACE** - ** = MDD** - **/ACE** - **
CTQ (*SD*)	68.5 (17.7)	39.2 (10.1)	60.9 (14.8)	30.8 (6.0)	*p* ˂ .001 MDD + /ACE + = MDD** - **/ACE + > MDD + /ACE** - ** > MDD** - **/ACE** - **
STAI-T (*SD*)	58.0 (8.8)	56.2 (9.7)	34.3 (7.9)	28.9 (6.5)	*p* ˂ .001 MDD + /ACE + = MDD + /ACE** - ** > MDD** - **/ACE + > MDD** - **/ACE** - **

*MDD* major depressive disorder, *ACE* adverse childhood experiences, *MDD* + */ACE* + MDD patients with ACE, *MDD* + */ACE*** - **MDD patients without ACE, *MDD*** - ***/ACE* + participants with ACE but no MDD, *MDD*** - ***/ACE*** - **participants without MDD and without ACE, *SD* standard deviation, *MADRS* Montgomery Asberg Depression Rating Scale, *BDI* Beck Depression Inventory, *ETI* Early Trauma Inventory, *CTQ* Childhood Trauma Questionnaire, *STAI*-*T* trait version of the State Trait Anxiety Inventory (trait sum score)

We found no significant differences between groups regarding sex, age, body mass index, and education level. In women only, we found no significant group differences in use of hormonal contraceptives, cycling phases between groups on both testing days (day 1: *χ*^2^ = 4.02, *df* = 6, *p* = 0.68; day 2: *χ*^2^ = 3.96, *df* = 6, *p* = 0.68) nor in state of menopause (yes/no) (*χ*^2^ = 1.51 *df* = 3, *p* = 0.68). Furthermore, clinical characteristics of MDD patients did not differ between MDD patients with and without ACE (MDD + /ACE + , MDD + /ACE -) in regard to age at MDD onset and length of current MD episode. However, time since MDD onset was longer in MDD patients with ACE (MDD + /ACE +), which may be explained by their slightly older age, even though not significant, at study participation. As expected and also presented in earlier publications of this research project (de Punder et al. 2018; Deuter et al. 2020; Kuehl et al. 2020; Schulz et al. 2021), MADRS and BDI sum scores were significantly higher in the MDD groups (MDD + /ACE - , MDD + /ACE +) compared to the two healthy groups (MDD - /ACE - , MDD - /ACE +). There were no significant differences in MADRS and BDI sum scores in between the MDD groups (MDD + /ACE - , MDD + /ACE +) and the healthy groups (MDD - /ACE - , MDD - /ACE +). In addition to the main diagnosis of current major depression, twelve MDD + /ACE + patients (phobia: *N* = 3, PTSD [related to ACE]: *N* = 5, personality disorders: *N* = 4, dysthymia: *N* = 1, bulimia nervosa: *N* = 1, somatoform pain disorder: *N* = 1) and six MDD + /ACE - patients (phobia: *N* = 2, personality disorders: *N* = 2, dysthymia: *N* = 1, somatoform pain disorder: *N* = 1) fulfilled the criteria for one or more mental comorbid disorders. As expected due to our recruitment criteria, ETI and CTQ sum scores were higher in the groups with ACE (MDD + /ACE + , MDD - /ACE +) compared to the groups without ACE (MDD + /ACE - , MDD - /ACE -): ETI sum scores were significantly higher in the ACE + groups (MDD + /ACE + , MDD - /ACE +) compared to the ACE - groups (MDD + /ACE - , MDD - /ACE -) with no significant differences within groups with ACE (MDD + /ACE + , MDD - /ACE +) and groups without ACE (MDD + /ACE - , MDD - /ACE -). Higher CTQ sum scores were observed in the ACE + groups (MDD + /ACE + , MDD - /ACE +) compared to the ACE - groups (MDD + /ACE - , MDD - /ACE -). Sum scores of the additionally assessed STAI-T (trait version of the State-Trait Anxiety Inventory (STAI-T; Laux et al. 1981)) were significantly higher in the two MDD groups (MDD + /ACE - , MDD + /ACE +) compared to the two healthy groups (MDD - /ACE - , MDD - /ACE +) with no significant differences within both MDD groups, but a significant higher averaged STAI-T score in the MDD - /ACE + group compared the MDD - /ACE - group.

### Physiological measures

**Fig. 2:**
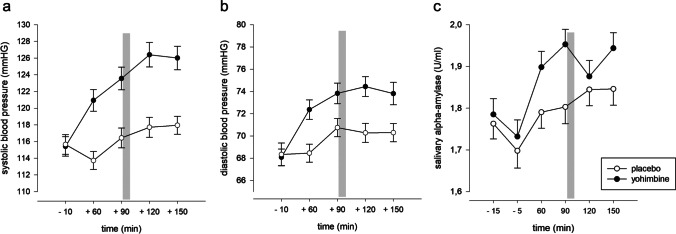
Systolic (2a), diastolic (2b) blood pressure, and salivary alpha-amylase (2c) over time with administration of placebo and yohimbine across groups. Grey bars indicate time window of the emotional dot probe paradigm. Error bars represent ± 1 SE.

All physiological measures showed a significant increase over time that was more pronounced following yohimbine compared to placebo as displayed by significant interaction effects of “time” and “treatment” for sAA (*F*_5,535_ = 10.06, *p* < 0.001) as well as for systolic (*F*_4,452_ = 20.64, *p* < 0.001) and diastolic blood pressure (*F*_4,422_ = 7.75, *p* < 0.001). Please see also Fig. 2. More detailed results are presented in Kuehl et al. (2020).

### Attentional bias

Reaction times that were less than 100 ms (anticipation error) or greater than 1500 ms (lack of concentration) were excluded from statistical analyses to reduce the influence of ouliers. This was the case for *N* = 3 (one MDD patient without ACE, two healthy participants without ACE). Only reaction times from correct responses were analyzed. Overall, data of three participants were excluded due to less than 50% correct responses on one or both test sessions, indicating insufficient comprehension of the task rules (MDD + /ACE - : *N* = 1, MDD - /ACE - : *N* = 2). The percentage of correct answers was on average 99% or above in all test conditions in all groups.

**Fig. 3:**
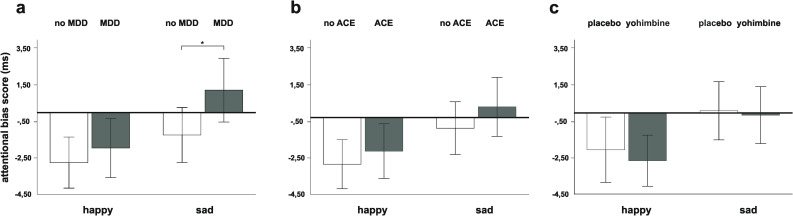
Attentional bias scores (reaction times (ms) of incongruent minus congruent trials with 95% confidence intervals indicated) toward and away from emotional face pictures (happy, sad) in the dot probe task according to (3a) major depressive disorder (MDD) across the factors “ACE” and “drug condition,” (3b) adverse childhood experiences (ACE) across the factors “MDD” and “ drug condition,” and (3c) experimental drug condition (placebo vs. yohimbine) across the factors “MDD” and “ACE”.

2 × 2 × 2 repeated measures ANOVAs with “treatment” (yohimbine vs. placebo) as within-subject factor and “MDD” (MDD vs. non MDD) and “ACE” (ACE vs. non ACE) as between-subject factors were conducted for happy and sad face pictures, separately. For happy face pictures, no significant main effects of “treatment” (*F*_1,124_ = 0.24, *p* = 0.624, partial eta^2^ = 0.002), “MDD” (*F*_1,124_ = 0.55, *p* = 0.461, partial eta^2^ = 0.004) or “ACE” (*F*_1,124_ = 0.50, *p* = 0.483, partial eta^2^ = 0.004) and no significant interaction effects were found. For sad face pictures, repeated measures ANOVA of bias scores revealed a significant main effect of “MDD” (*F*_1,124_ = 4.45, *p* = 0.037, partial eta^2^ = 0.035), indicating that MDD patients directed more attention to sad facial expressions (please see Fig. 3). There were no significant main effects of “treatment” (*F*_1,124_ = 0.05, *p* = 0.828, partial eta^2^ < 0.001) or “ACE” (*F*_1,124_ = 1.14, *p* = 0.289, partial eta^2^ = 0.009) and no significant interaction effects. The results did not change, when we controlled for drug order (placebo/yohimbine). In addition, there was no effect of test session order.

## Discussion

To investigate the potential influence of the noradrenergic system on a negative attentional bias in individuals with major depression and childhood adversity, we tested the effect of enhanced noradrenergic activation induced by acute administration of the alpha2-receptor blocker yohimbine on attentional biases. An emotional dot probe task served as a measure of attentional bias. We further aimed to investigate biased attention influenced by NA activity as a potential mechanism for how ACE contribute to vulnerability to and maintenance of MDD.

We tested different hypotheses, investigating the effects of MDD on biased attentional processing per se and the possible influence of noradrenergic stimulation and ACE. These hypotheses have been partially confirmed. Our results show a significant effect of MDD when attentional bias scores for sad face pictures were analyzed. This could be carefully interpreted as facilitated attentional processing of sad emotional pictures in MDD patients (compared to non-MDD individuals), consistent with the hypothesis of a negative attentional bias in patients with MDD. Contrary to what was originally hypothesized, this effect was neither significantly increased by ACE nor decreased by the administration of yohimbine; these factors did not affect attentional processing.

An attentional bias of MDD patients for sad, but not happy faces in comparison to non-MDD, has been described before, especially as difficulty in disengaging attention from negative stimuli (Armstrong and Olatunji 2012; Caseras et al. 2007; Eizenman et al. 2003; Mathews and MacLeod 2005). Of note, we found no differences in attention bias scores of happy faces between MDD patients and non-MDD participants. This is in contrast to our hypothesis of an attentional bias away from positive face stimuli in MDD patients compared to non-MDD participants. A meta-analysis (Winer and Salem 2016) provides evidence for avoidance of positivity in depressed patients. The authors interpret this as arising from reward devaluation, rather than from initial lack of reward valuation. However, many dot-probe studies did not find effects of MDD for happy stimuli (e.g., (Gotlib et al. 2004; Zhou et al. 2015)). Interestingly, in a systematic investigation series regarding the dot probe paradigm in healthy participants, Puls and Rothermund (Puls and Rothermund 2018) demonstrated no evidence for a general effect for emotional faces in dot probe tasks for healthy participants, leading to the conclusion that individuals do not attend automatically to emotional faces. Furthermore, the authors state that attentional capture for specific emotional stimuli should be much more likely to occur if these stimuli are made relevant for the participants. Our results are in line with the assumption that only sad faces served as relevant stimuli primarily for MDD patients and captured their attention, thereby leading to an attentional bias toward sad faces. Happy faces might not have been relevant enough for MDD patients, and neither happy nor sad faces might have been relevant enough for healthy individuals, so no bias toward those pictures occurred. A mood congruent bias of MDD patients toward sad faces could be thus explained. However, previous studies using dot probe paradigms usually found a mood-congruent attention bias in MDD patients only when longer stimuli exposures than in our study were used (Joormann and Quinn 2014; LeMoult and Gotlib 2019). While MDD in itself biased attentional processing of sad facial stimuli, we did not find an effect of yohimbine administration or a history of childhood trauma on attentional bias.

An absence of the yohimbine effect raises the question of wether the dose of administered yohimbine was high enough to raise noradrenergic activity to an effective level. Indeed, our dose was lower compared to other studies that found effects on cognitive processes (e.g., O'Carroll et al. 1999; Soeter and Kindt 2011, 2012). However, in a study by Wingenfeld et al. (Wingenfeld et al. 2013), only 5 mg were administered, yet this still had an effect on memory. Furthermore, an increase in blood pressure and saliva alpha amylase indicates a sufficient effect on noradrenergic activity. However, these are measures from the periphery, and in the absence of any direct measure of central effects, we cannot make a final statement about the actual central NA activity.

Another explanation might be that noradrenergic activation simply has no effect on attentional bias for emotional face stimuli. However, a study by Vasa et al. (Vasa et al. 2009) found an effect of yohimbine administration, in that a bias toward angry faces under placebo was absent under yohimbine. Still, that study investigated only healthy participants and used other emotional facial expressions as stimuli compared to our study. Since we tested only sad and happy, but not angry or any further emotional facial expressions in our paradigm, we can only speculate about the specific role of distinct emotions for the effect of noradrenergic activity. As increased noradrenergic activity, as for example resulting from yohimbine administration, has been linked to anxiety and fear (for review Bremner et al. 1996a, b; Tanaka et al. 2000)), anxiety-related emotional stimuli such as fearful or angry faces might be more affected. In a series of studies (Harmer et al. 2003; Harmer et al. 2004; Murphy et al. 2009) that compared effects of selective serotonin reuptake inhibitors (SSRI) versus selective noradrenaline reuptake inhibitors (SNRI) on several cognitive biases in healthy participants found overall increased processing of positive material compared to negative material for both antidepressant drugs. However, there was no effect of a noradrenergic challenge by SNRI on attentional bias for fearful faces, but of SSRI based on a serotonergic mechanism (Murphy et al. 2009). Overall, the authors suggest a more depression specific effect of a noradrenergic intervention with SNRI, while SSRIs seem to work for anxiety-related material as well. Of note, two of the studies (Harmer et al. 2004; Murphy et al. 2009) used a short-term intervention of seven days and not an acute single drug administration as in our study.

While we found neither an effect of ACE nor an interaction effect of ACE x MDD, some studies indicate associations between attentional biases for emotional material and ACE (Bodenschatz et al. 2019; England-Mason et al. 2018; Günther et al. 2015; Wells et al. 2014). However, most of these studies found rather specific ACE-related associations such as in postpartum women with greater experiences of maltreatment and difficulties with emotion regulation (England-Mason et al. 2018) or under cognitive load condition solely (Wells et al. 2014). In the study of Bodenschatz et al. (Bodenschatz et al. 2019), ACE were associated with reduced attention for angry and sad facial expressions in depressed patients. As we only used sad facial expressions as negative stimuli, we can only speculate about an emotion specific attentional biases in MDD patients with ACE. In Günther et al.’s study (Günther et al. 2015), however, attentional bias toward sad facial expressions was positively correlated with severity of ACE in MDD patients. In contrast to our study, a longer stimulus duration of 1000 ms was used. As earlier studies found attention bias in MDD patients only with longer stimuli durations (Joormann and Quinn 2014; LeMoult and Gotlib 2019), this might also explain the discrepancy between the results. While our results cannot support an effect of ACE on attention bias, our results are in line with a study of Vrijsen et al. (Vrijsen et al. 2014) that investigated associations between ACE, biased information processing and depression onset and recurrence in a large sample of formerly depressed individuals, and found no evidence for an association between ACE and depression-related biased information processing. Taken together, the existing body of research provides insufficient information to explain fully how an attentional bias in MDD patients might be related (or not) to a history of ACE.

Of note, the reliability of the dot probe task has been subject to criticism. In his work, Schmukle (Schmukle 2005) fundamentally questioned the validity of the results and the test–retest reliability of the dot probe paradigm in non-clinical studies. He repeatedly administered the same task to a sample of healthy participants and found substantially divergent results. Further studies produced null results or found a lack of reliability as well, indicating an absence of emotional validity effects in the classical dot probe task using emotional words of facial expressions (Chapman et al. 2019; Puls and Rothermund 2018; Staugaard 2009). Similar problems exist with other behavioral response tasks that build on attentional allocation to task-irrelevant emotional faces (Tannert and Rothermund 2020; Victeur et al. 2020). This must be taken into account when interpreting our results. However, it is of importance that the studies criticizing the properties of the task were conducted with healthy participants. In clinical populations, the effects of emotional stimuli, with their ability to capture and bind attention, may be dissimilar and more pronounced (Yiend 2010). This again is also supported by the numerous previous studies listed in the introduction, which were able to demonstrate aberrant response patterns in patients with MDD (Armstrong and Olatunji 2012; Caseras et al. 2007; Mathews and MacLeod 2005; Peckham et al. 2010).

### Limitations

The results of our study must be interpreted with caution due to some limitations. Firstly, our emotional stimuli material consisted of happy and sad facial expression only, which limits the comparability between different emotions. Especially for effects of ACE and NA effects, a further comparison with angry and fearful facial expressions as additional face stimuli would have been of great interest. Additionally, we only used short stimuli durations. Interestingly, this is the first study that could demonstrate an attentional bias in MDD patients even with short stimulus duration of 500 ms. However, we cannot exclude that effects of ACE and yohimbine might be found with longer stimuli durations. Even though a negative attentional bias has been more consistently found in MDD when face pictures compared to words were used as stimuli (for review (LeMoult and Gotlib 2019)), it is possible that using more personally relevant stimuli could have increased subjective salience and thus overall attentional vigilance toward the stimuli, which in turn might have led to detection of an effect in individuals with ACE. Furthermore, we did not use different dosages of yohimbine, which limits conclusions about potential dose–response-relations between noradrenergic activity and effects on attentional bias. Even though the results for blood pressure and salivary alpha-amylase indicate an increase in noradrenergic activity after yohimbine intake, it should be noted that these are only indirect measures of potential NA effects on a central level. 

## Conclusions

In summary, our study results are consistent with the hypothesis of a negative attentional bias in patients with MDD. In contrast to our hypotheses, we found no association of negative attentional bias with childhood adversity or with an acute stimulation of the noradrenergic system. Further research is needed to understand by which mechanisms childhood adversity increases the risk for MDD and to understand the biological basis of the MDD-related negative attentional bias.
